# Development and preliminary evaluation of a ciliary muscle–oriented motor imagery script for primary school children: a school-based cluster randomized controlled trial

**DOI:** 10.3389/fpsyg.2026.1809541

**Published:** 2026-05-07

**Authors:** Meng Zhang, Sheng Zhou, Xu Zhang

**Affiliations:** 1 Department of Basic Courses, Suzhou City University, Suzhou, China; 2 Sport Department, Suzhou University of Technology, Suzhou, China

**Keywords:** children, ciliary muscle regulation, imagery ability, motor imagery, PETTLEP model

## Abstract

**Background:**

Myopia in children is occurring earlier and progressing faster, while existing interventions such as outdoor activity and device-based visual training are often difficult to implement consistently in school settings. Motor imagery (MI), as a low-burden cognitive approach, may offer a complementary pathway for visual-function support. However, ciliary muscle–oriented MI scripts specifically designed for children remain underdeveloped.

**Purpose:**

This study aimed to develop a ciliary muscle–oriented MI script based on the PETTLEP model for primary school children and to compare two training arrangements: G1 (staged imagery group: 8 weeks of general imagery followed by 8 weeks of specific imagery; *n* = 76) versus G2 (specific-only group: 16 weeks of specific imagery; *n* = 80).

**Methods:**

A school-based cluster randomized design was used. A total of 156 children aged 10–11 years from one primary school in Suzhou were assigned by intact class to either G1 or G2. Visual outcomes, including uncorrected distance visual acuity (UDVA), kinetic visual acuity (KVA), accommodative amplitude (AMP), and binocular accommodative facility (BAF), were assessed at baseline (T1) and post-intervention (T3). Imagery ability was assessed using the Sport Imagery Questionnaire (SIQ; cognitive-general imagery [CG] and cognitive-specific imagery [CS] dimensions) at T1, T2, and T3.

**Results:**

Both groups improved over time on several outcomes. No between-group difference was found for UDVA at T3. After Benjamini–Hochberg correction, KVA was the only visual outcome showing a significant adjusted between-group difference at T3; BAF was not significant at T3, and AMP showed only a borderline trend. Significant Time × Group interactions were found for KVA, BAF, and CG. G1 scored higher than G2 on CG at both T2 and T3. In CS, both groups improved over time; G2 scored higher at T2, but no between-group difference was found at T3. However, because the study included only four classes in total (two per condition) and the class-level ICC for post-intervention KVA was 0.366, the KVA finding should be interpreted as a hypothesis-generating class-level observation rather than a reliable training effect.

**Conclusion:**

This study developed and preliminarily evaluated a ciliary muscle–oriented MI script under structured school-based conditions. The clearest between-group evidence was observed for CG. Different imagery arrangements may be associated with different developmental patterns, but visual-function findings, particularly KVA, require cautious interpretation and replication in studies with more clusters and stronger control conditions.

## 1 Introduction

Visual function issues in children have emerged as a significant public health concern, affecting adolescents’ health and academic performance. The prevalence of myopia has continued to rise in recent years, trending toward earlier onset and faster progression, making it one of the most common eye health problems worldwide across a wide range of ages (Baird et al., 2020; Yusuf and Wang, 2020; Gupta et al., 2021). Notably, in East Asia, childhood myopia prevalence has exceeded 50% and continues to increase at a significantly faster rate than in Caucasian children (Recko and Stahl, 2015). This difference remains statistically significant after controlling for genetic factors, suggesting that environmental factors play a crucial role in the development of childhood myopia. In addition to reduced visual acuity, myopic children frequently exhibit accommodative dysfunction, decreased kinetic visual acuity (KVA), and visual fatigue. A substantial proportion of school-related learning activities rely on visual function (Narayanasamy et al., 2016); consequently, inadequate visual function can negatively affect reading, attention, and academic performance.

Schools, as the primary settings for children’s daily learning and living, are an important context for delivering structured myopia prevention and control strategies. School-based behavioral and environmental interventions, such as increasing physical activity duration, improving lighting conditions, and regulating visual habits, have been shown to help slow the onset and progression of myopia at the population level, and are commonly included in current prevention strategies. However, existing interventions have notable practical limitations. While outdoor physical activity has demonstrated protective effects against the progression of myopia in children, existing research has not clearly disentangled the respective roles and mechanisms of physical activity and outdoor light exposure (Zhou et al., 2023). Furthermore, outdoor activity interventions are highly dependent on time, space, and weather conditions, which may limit their consistent implementation and contribute to variability in individual adherence. Conversely, most existing visual training approaches rely on specialized equipment or passive stimulation, which are difficult to integrate into routine school instruction. Research suggests that the effectiveness of physical activity in preventing myopia depends on the effective combination of external visual stimulation and self-directed visual regulation (Tao, 2023). However, visual tasks in school-based physical activities are predominantly externally driven, which may be insufficient for systematically cultivating children’s autonomous visual regulation abilities. Therefore, it is important to explore low-burden, classroom-compatible, cognitively oriented complementary intervention pathways that can be feasibly integrated into routine school settings.

Among the physiological mechanisms underlying visual function, the ciliary muscle plays a central role. It is directly involved in accommodation of the crystalline lens, and its functional status is closely associated with accommodative amplitude (AMP), accommodative facility, and accommodative lag—factors that are linked to visual clarity, visual stability, and the risk of myopia progression. According to accommodation theory, changes in the eye’s focal length primarily depend on the contraction and relaxation of the ciliary muscle, which modulates the tension of the zonular fibers to alter lens curvature and enable switching between near and distant focus (Schachar, 2006; Ovenseri-Ogbomo and Oduntan, 2015; Lu et al., 2021). Although the ciliary muscle is biologically classified as smooth muscle, its ultrastructural and neural innervation characteristics suggest a relatively high degree of neural modifiability (Flügel et al., 1990), providing a psychological and physiological basis for the potential cognitive modulation of accommodation.

Psychoneuromuscular theory and the functional equivalence hypothesis suggest that motor imagery (MI) can partially recruit neural and physiological processes that overlap with those involved in actual performance. In visual-function contexts, this provides a rationale for exploring whether imagery-based practice may support accommodative regulation and related outcomes such as uncorrected distance visual acuity (UDVA) and KVA. This rationale is potentially relevant to the ciliary muscle, which plays a central role in accommodation. Although the ciliary muscle is an intraocular smooth muscle and does not provide readily accessible conscious proprioceptive feedback, MI has been shown to engage brain regions involved in actual movement and to elicit autonomic responses proportional to imagery intensity (Fusi et al., 2005). Pearson and Westbrook (2015) further described imagery-related experience as a form of “phantom perception,” suggesting that visual experience is not entirely dependent on immediate retinal input.

Additional support comes from research indicating that imagery can influence autonomically regulated ocular and perceptual processes. For example, conscious imagery can induce changes in pupil diameter (Rozado et al., 2019), and imagined changes in luminance can evoke pupil responses resembling those observed under actual illumination changes (Laeng and Sulutvedt, 2014). More broadly, depictive accounts of mental imagery propose that imagery is a top-down process grounded in prior perceptual experience and partially shares mechanisms with perception (Kosslyn and Shwartz, 1977; Currie, 1995). Taken together, these findings support the possibility that ciliary muscle-oriented imagery may be conceptualized not as direct perception of muscle contraction or relaxation, but as imagery of visual-adjustment-related experiences, such as changes in clarity, ocular effort, and accommodative transition. At the same time, existing MI-related work in pediatric visual function remains limited and methodologically heterogeneous, underscoring the need for more structured and child-appropriate imagery scripts.

Based on these considerations, the present study had two aims. First, we sought to develop a ciliary muscle-oriented motor imagery (MI) script for primary school children based on the PETTLEP model. Second, we compared two school-based training arrangements: Group 1 (G1; staged imagery group) and Group 2 (G2; specific-only group). We hypothesized that both arrangements would be associated with improvements over time in imagery ability and selected visual-function outcomes. We further hypothesized that the staged G1 arrangement might be associated with a more favorable pattern of change than the G2 arrangement, particularly for imagery-related outcomes.

## 2 Materials and methods

### 2.1 Design and participants

This study was a school-based cluster randomized controlled trial (RCT) with the class as the unit of randomization (cluster) and the individual child as the unit of measurement and inference. The trial was conducted in one primary school in Suzhou, China, within routine school schedules.

A total of four intact classes were included. Two classes (classes 4.2 and 4.5; *n* = 76; mean class size = 38.0) were allocated to Group 1 (G1; staged imagery group), and two classes (classes 4.1 and 4.6; *n* = 80; mean class size = 40.0) were allocated to Group 2 (G2; specific-only group). In G1, participants received 8 weeks of general imagery followed by 8 weeks of specific imagery, whereas in G2, participants received 16 weeks of specific imagery. Cluster randomization was performed at the class level using a lottery procedure. The allocation process was conducted by a staff member not involved in outcome assessment or intervention delivery, to reduce allocation bias. Because the study used class-level randomization within one school, formal allocation concealment was limited. To minimize allocation bias, the lottery-based randomization was conducted by an independent staff member who was not involved in outcome assessment or intervention delivery. Outcome assessors remained blinded to group allocation throughout data collection and preliminary data processing, although facilitators necessarily became aware of group assignment for intervention delivery.

Visual-function outcomes, including UDVA, KVA, AMP, and binocular accommodative facility (BAF), were measured at baseline (T1) and at week 16 (T3; week 16). Imagery-ability outcomes, including CG and CS, were assessed at baseline (T1), mid-intervention (T2; week 8), and post-intervention (T3; week 16). Assessors responsible for visual function and questionnaire measurements did not participate in the delivery of the intervention and remained blinded to group allocation throughout data collection and preliminary data processing. Participants, teachers, and outcome assessors were not informed of the specific study hypotheses, whereas facilitators were aware of the training procedures required to administer the intervention. The final analytical sample comprised 156 primary school children from four intact Grade 4 classes at one primary school in Suzhou, China. All participants were aged 10–11 years at baseline, and the sample included 74 boys and 82 girls. Baseline visual-function characteristics are presented in Table 1, and no significant between-group differences were observed at baseline (all *p* > 0.05).

**Table 1:** Participants’ baseline visual function indicators.

Group (*N*)	UDVA (Left)	UDVA (Right)	KVA	BAF	AMP
G2 (80)	4.64 ± 0.39	4.59 ± 0.38	0.35 ± 0.25	8.07 ± 2.07	10.19 ± 2.81
G1 (76)	4.69 ± 0.38	4.64 ± 0.42	0.34 ± 0.24	7.85 ± 2.20	10.35 ± 3.00
*t*	0.799	0.857	−0.389	−0.643	0.327
*p*	0.397	0.284	0.611	0.964	0.732

Inclusion criteria:

1. Aged 10–11 years;
2. No astigmatism, no hyperopia, no history of orthokeratology lens wear, and no other pathological eye diseases;
3. Normal cognitive function, with the ability to understand and follow verbal instructions to complete training and assessment tasks.

Exclusion criteria:

1. Binocular UDVA or corrected visual acuity below 4.0;
2. Presence of pathological eye diseases;
3. Inability to complete visual function measurements or ocular biometric assessments;
4. Incomplete or invalid questionnaire responses (i.e., one or more unanswered items, or response patterns exhibiting uniform or clearly patterned selections);
5. Incomplete participation in the intervention due to uncontrollable factors such as school transfer.

It should be noted that the G1 and G2 conditions differed simultaneously in two aspects: the type of imagery content delivered and the sequence in which the training phases occurred. Consequently, the present design does not allow the independent effects of imagery content and training sequence to be fully disentangled. Any observed differences between the two groups may therefore reflect a combination of content-related and sequencing-related influences.

### 2.2 Outcome measures

To ensure the experiment’s scientific rigor and reliability, a series of standardized instruments and testing procedures were used throughout the intervention. Visual function outcomes included UDVA, KVA, AMP, and BAF. The assessment procedures for UDVA, KVA, and BAF followed previously established standardized methods described in our prior study (Zhou et al., 2025), in which the corresponding instruments and testing procedures were reported in detail. Imagery ability was assessed using the CG and CS subscales of a modified version of the Sport Imagery Questionnaire (SIQ). The questionnaire was adapted in our previous study to reflect children’s visual imagery tasks in classroom settings and demonstrated acceptable reliability and construct validity in a child sample (Zhou et al., 2025).

AMP was measured using an accommodative ruler. During the test, participants wore their optimal refractive correction and were asked to binocularly fixate on a visual target positioned at 40 cm along the best visual acuity line. The accommodative ruler was aligned with the participant’s midline at the level of the nasal bridge. Participants were instructed to maintain fixation on the target and report any changes in its clarity, while the examiner simultaneously observed and recorded ocular movements (Burns et al., 2020; Momeni-Moghaddam, et al., 2014).

The push-up and push-away methods were combined to measure AMP. During testing, the examiner moved the visual target toward the participant’s eyes at a constant speed of 2 cm/s until the participant reported that the target began to blur. The distance between the target and the spectacle plane was then recorded. The target was subsequently moved away from the participant until it was reported that it had become clear again; the distance was recorded. The near-point and far-point distances were recorded, and accommodative amplitude was calculated as the dioptric difference between the reciprocals of these two distances.

The formula for calculating AMP was as follows:


$$\begin{array}{l} \mathrm{AMP}\;(D)=\frac{1}{\text{near}-\text{point distance}(m)}\\ -\frac{1}{\mathrm{far}-\text{point distance}(m)} \end{array}$$ (1)

### 2.3 Statistical analyses

Because children were nested within classes, intervention effects were analyzed using linear mixed-effects models (LMMs) with a random intercept for class to account for intraclass correlation (Wang et al., 2026; Billot et al., 2024). For each outcome, the post-test score was modeled as a function of group and the corresponding baseline score:


$$\text{Post}\sim \beta_{ο}+\beta ₁\cdot \text{Group}+\beta ₂\cdot \mathrm{Pre}+u\_j+\varepsilon$$ (2)

*u_j ~ N(0, σ^2^_u)* represents the class-level random intercept, and *ε ~ N(0, σ^2^_e)* represents the individual-level residual error. Model parameters were estimated using restricted maximum likelihood (REML), and Wald 95% confidence intervals were reported for fixed-effect estimates.

Class-level intraclass correlation coefficients (ICCs) were estimated from null random-intercept models as *ICC = σ^2^_cluster / (σ^2^_cluster + σ^2^_residual)* and are reported in Supplementary material.

To control multiplicity across the six outcomes, raw *p*-values for the primary between-group intervention effects were adjusted using the Benjamini–Hochberg (BH) procedure to control the false discovery rate (FDR = 0.05). Effect sizes were reported as Cohen’s d (between-group mean difference divided by the pooled standard deviation). All analyses used two-sided tests with *α* = 0.05. Statistical analyses were conducted in Python using the statsmodels package (MixedLM).

### 2.4 Guided imagery script development

The effectiveness of MI training primarily relies on the quality of the guided imagery script. High-quality MI training requires that imagery content be clear, complete, and stable to support a continuous, coherent, and repeatable imagery process, thereby facilitating the consolidation and transfer of training effects. For MI interventions aimed at improving children’s visual function, the scientific and standardized design of guided imagery scripts is particularly critical, as it influences whether children can form clear mental images and whether they can effectively engage visual attention and the physiological responses associated with accommodation.

In this study, a ciliary muscle–oriented motor imagery (MI) script served as the primary intervention. We systematically developed and conducted a preliminary evaluation of a guided imagery script designed for primary school children under structured classroom conditions. The script was grounded in the PETTLEP model of MI and integrated principles from the functional equivalence hypothesis and psychoneuromuscular theory to ensure the clarity, specificity, and stability of the imagery process. The PETTLEP model, proposed by Holmes and Collins (2001), comprises seven elements: Physical, Environment, Task, Timing, Learning, Emotion, and Perspective. It aims to maximize functional equivalence between imagery and actual performance, and therefore provides a useful framework for constructing child-oriented imagery scripts that are context-specific, emotionally congruent, and task-relevant.

#### 2.4.1 5W framework for script design

The imagery script serves as the primary medium through which participants perceive MI, and its quality directly influences the feasibility and effectiveness of training. Williams et al. (2013b) proposed that the development and implementation of MI scripts should follow a systematic 5 W framework—Who, Where and When, Why, What, and How—to ensure that the script content aligns closely with user characteristics, application contexts, and intervention objectives. Therefore, during the planning stage of script development, this study strictly applied the 5 W framework to systematically define and optimize the target users, training context, functional objectives, imagery content, and presentation format.

The script was designed for primary school children aged 10–11 years. At this developmental stage, children’s imagery ability is developing rapidly, but their attention span remains limited, necessitating a balance between vividness and cognitive load. We therefore ensured that the script’s language was as concrete, vivid, and visually descriptive as possible, with gradually increasing complexity to accommodate children with varying levels of imagery ability. We adopted a multimodal approach to presentation, combining video, audio, images, and physical objects.

Improvements in visual function are typically gradual and cumulative, requiring sufficient time to induce neuroplastic changes and enhance accommodative capacity. Therefore, the intervention period was set at 16 weeks, with three sessions per week. This design was informed by our previous longitudinal studies on cognitive training and MI interventions in children. At the same time, the schedule accounted for primary school students’ academic workload and cognitive load tolerance, ensuring practical feasibility and compliance. Regarding session duration, Munzert et al. (2009) suggested that effective MI training should typically be limited to 5–15 min per session to maintain the integrity of the imagery experience and the concentration of cognitive processing. Similarly, Weinberg (2008) recommended that imagery training for children avoid sessions shorter than 5 min, which may be ineffective, and sessions longer than 15 min, which could induce mental fatigue and attentional distraction. Therefore, given children’s attention spans and the practical requirements of imagery training, each session in this study was limited to 5–10 min, thereby balancing training effectiveness with the risk of cognitive fatigue from excessive duration. The script was implemented in school classroom settings in small-group formats and scheduled during physical education classes or extended recess periods. The training environment was kept relatively quiet, and soft lighting was employed to minimize external distractions and facilitate focused engagement in the imagery state.

#### 2.4.2 Imagery materials

Participants were unfamiliar with both the content and methods of imagery training and had limited exposure to similar practices in daily life. Therefore, the design of general imagery materials may be particularly important for improving children’s visual function. Paivio (2008) proposed that imagery training influences motor performance through cognitive and motivational functions: general cognitive imagery primarily enhances cognitive processing, strategic thinking, and overall skill representation, whereas specific cognitive imagery focuses on simulating particular movements and refining skill execution. Based on this theoretical framework, the design of MI materials should be aligned with children’s cognitive developmental level and practical needs, ensuring that initial-stage general imagery training lays a solid foundation for subsequent skill-specific imagery. We selected general imagery materials based on simulations of everyday experiences and natural scenes to help children understand MI training and construct detailed representations of visual tasks, thereby optimizing visual-cognitive function (Smith, 2007). These materials were designed to engage children visually and emotionally, to construct a broader visual and psychological context, to strengthen their ability to form mental imagery, and to prepare them for more advanced visual tasks and specific imagery training. Examples of general imagery materials are presented in Table 2.

**Table 2:** Examples of general imagery materials.

Scenario	Materials (Imagery Content)	Purpose
Natural scenes	A quiet forest, a gently flowing stream, or a deserted beach; sunlight, blue sky, and white clouds; standing by the seaside looking at oneself on the shore, then raising the head to watch a boat approaching from afar; birds flying in the sky.	Relaxation and stress relief
Daily life	Walking on the way home or playing with family; distant buildings, nearby trees; sitting on the sofa in the living room, noticing the layout of the room, the position of furniture, and paintings on the wall; distant television and a cup of tea on the nearby table.	Focused attention and emotional engagement
Learning contexts	A cup, book, or pen on the desk in the classroom; chalk marks on the blackboard, the teacher standing in front, desk arrangement, scenery outside the window.	Concentration and attention enhancement
Sports contexts	One’s favorite sport or physical education class, such as cycling, football, or swimming; a park, distant amusement facilities, nearby sports equipment.	Enhanced body awareness
Others	A ball rolling along a straight line while the eyes follow its trajectory; toys of different sizes in a toy box, wooden blocks, geometric shapes; a large red circle and a small blue square.	Perception of distance, size, shape, and color

In MI training, specific imagery differs from holistic imagery: rather than broadly imagining an entire sequence of movements or the overall context of a sports scenario, specific imagery focuses on imagining a particular motor action, such as a basketball shot or a soccer kick. In the present study, specific imagery materials involved dynamic visual tasks featuring forward–backward movement extracted from sports activities. Unlike general imagery, which primarily emphasizes cognitive abilities and the quality of mental imagery, specific imagery focuses on concrete visual tasks and aims to improve visual function and ocular muscle regulation through precise simulation.

In the context of improving children’s visual function, the specific imagery materials were designed to activate and train the ciliary muscle through dynamic near–far visual tasks, including alternating near–far visual tracking, focusing, and depth-perception training. These dynamic visual tasks simulate real-life distance-switching situations, optimize the eye’s ability to shift between different visual distances, and help children enhance accommodative function. Three-dimensional (3D) visual training is based on similar principles and has been shown to improve binocular accommodation and to have the potential to slow the progression of myopia (Chassine et al., 2015; Teng et al., 2022). Stereoscopic and moving visual targets facilitate eye movements, alleviate ciliary muscle spasm, and reduce accommodative lag caused by prolonged near work. Huang et al. (2022) demonstrated that 3D visual training can effectively improve binocular accommodative deficits in myopic individuals.

**Figure 1:**
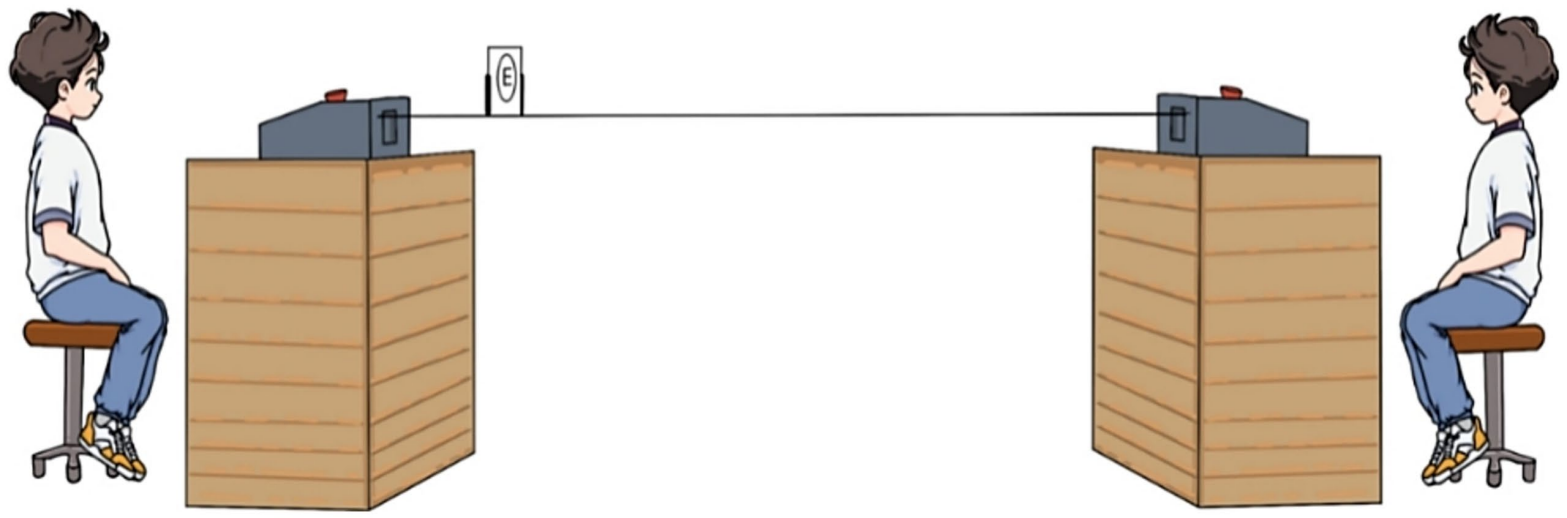
Specific imagery encoding task.

In this study, specific imagery centered on a near–far switching task involving a moving visual target at a distance of six meters. Children were instructed to close their eyes and imagine the visual target moving cyclically from far to near and from near to far. This process is intended to approximate real accommodative conditions and may conceptually reflect the contraction–relaxation pattern of the ciliary muscle. Accordingly, it can be conceptualized as a task-specific, ciliary muscle–oriented imagery module, although its functional equivalence to actual accommodative processes requires empirical validation. The process of developing the specific imagery materials is depicted in Figure 1 and described in detail in previous work (Zhou et al., 2025).

#### 2.4.3 Content construction

We formulated the guided imagery instructions based on factors influencing imagery training to improve children’s visual function, as well as on the characteristics of materials used during the encoding phase. These were primarily grounded in the PETTLEP model of MI and embedded within multiple theoretical frameworks to ensure the clarity and stability of the imagery process. By incorporating into the instructions elements that simulated real-life tactile sensations, sounds, and emotions, the training aimed to ensure that children’s mental imagery was as close as possible to actual experience, thereby enhancing its vividness and stability.

Establishing scenarios similar to the real training environment or using contexts familiar to children was intended to enhance immersion and realism during imagery. This approach enables children to generate clearer mental images and maintain more stable attention during imaginative tasks, thereby reducing their susceptibility to external distractions.

Given that shifts among imagery perspectives—internal visual imagery (IVI), external visual imagery (EVI), and kinesthetic imagery (KI)—may all be involved in ciliary muscle MI, we designed both general and specific guided imagery scripts that incorporated different perspectives and elements. These are summarized in Table 3.

**Table 3:** Examples of guided imagery instructions.

**Elements**		**Example of guided imagery instructions**	**Purpose**
Imagery type	General imagery	“Imagine you are sitting in your classroom. Lower your head and look at the books and stationery on your desk. Then lift your head and notice the words your teacher has written on the blackboard.”	Relaxation and retrieval of familiar imagery contexts
	Specific imagery	“Imagine that you are beginning your visual training task. Get ready. Focus on the near visual target. Start. The target moves slowly away (pause for 3 s), then stops (pause for 3 s). Get ready. Focus on the far visual target. Start. The target moves slowly toward you (pause for 3 s), then stops (pause for 3 s).”	Enhance awareness of clear vision by imagining detailed distant objects
Imagery factors / Perspective	External imagery (EVI)	“This is the first time you see the moving target. The teacher asks a student to sit down and demonstrates how to use it. You watch as the student places the visual target card on the moving track and begins to observe its movement.	Facilitate understanding of imagery content, strengthen memory, and reinforce visual-cognitive components
	Internal imagery (IVI)	“You see the moving track. Slowly walk closer, adjust your position, and sit down. Choose a visual target card that suits you, place it on the moving track, and begin to observe its movement.”	Enhance movement sensation and sense of presence, reinforcing visual-cognitive components
	Kinesthetic imagery (KI)	“You are looking at the visual target. It moves away and stops.” Blink your eyes. Then it moves toward you and stops. You can clearly imagine the feeling of your eyes focusing on the target. Open your eyes—the target appears in front of you. As you focus on the target, try to notice whether your eyes feel any different. You might notice changes in clarity, effort, or comfort. If you do not feel anything obvious, that is normal too.”	relax the ciliary muscle, and reduce ciliary muscle fatigue
PETTLEP	Physical	“You sit in a familiar position with your right hand resting on the console button. Feel the sensation of your fingertips. Press the button—you can hear the sound of the moving device starting…”	Arousal and attentional focus
	Environment	“You walk into the materials room, see the familiar moving track, walk to the green chair, and sit down…”	Increase imagery vividness
	Task	“Imagine your gaze following the moving visual target. Carefully observe its color, size, and shape, and try to see every detail clearly.”	Sustain focused attention
	Timing	“The target stays in front of you. Count: 1, 2, 3, then it continues moving away…”	Improve imagery accuracy
	Learning	“You are learning to track the visual target as it moves back and forth. With each practice, your gaze becomes more stable and more accurate in following the target.”	Strengthen visual cognition and motivation
	Emotion	“Each time you successfully complete a visual task, tell yourself: ‘I did very well! The target is becoming clearer in my eyes.’“	Enhance motivation, engagement, and maintain positive attitude

#### 2.4.4 Standardization procedure

Japanese scholar Satoshi Takano and colleagues developed a Motor Imagery Training Program (MITP) in 1995 (Liu, 2001), proposing 10 fundamental steps, including preparation, imagery elicitation, imagery inspection (internal and EVI practice), imagery movement (kinesthetic imagery practice), imagery experience, imagery control, imagery generation, imagery recall, imagery rehearsal, and synchronization with imagery practice. These steps were organized into three components: a foundational component, an emotional-experience component, and a technique-application component. MITP encompasses multiple aspects of imagery, such as perspective, arousal methods, temporal frequency, and imagery types, which are essential for enhancing the vividness and clarity of imagery and strengthening imagery control. However, this program is better suited to professional athletes with established competitive skills and experience.

Cumming and Quinton (2022) and Cumming et al. (2023) emphasized the need to transform MI interventions into standardized, comprehensive, and accurately reported protocols that clearly specify intervention procedures, adjustments, and guided instructions. The Template for Intervention Description and Replication (TIDieR), a checklist for reporting interventions, has been proposed to improve the effectiveness and scientific rigor of intervention descriptions and is applicable to MI training. The TIDieR checklist comprises 12 items: intervention name, rationale, materials, procedures, intervention provider, delivery mode, setting, dosage (time and intensity), personalization, modifications, and expected and actual outcomes (Dijkers, 2021).

In the present study, we used the TIDieR checklist to report the MI training intervention aimed at improving children’s visual function (Table 4). This approach was intended to clarify the fundamental principles, logical framework, and specific content of imagery training aimed at enhancing children’s visual function, ensuring the effectiveness and scientific validity of each component, and providing a foundation for the future dissemination of MI interventions for myopia prevention in children.

**Table 4:** Checklist of motor imagery training intervention for improving children’s visual function.

**Checklist item**	**Reported content/Description**
Item 1. Name: A brief name or phrase describing the intervention.	Motor imagery training for improving children’s visual function
Item 2. Why: The rationale, theory, or objectives underpinning the essential components of the intervention.	The application of motor imagery training may provide children with additional opportunities to promote visual health, strengthen the protective role of physical activity in myopia prevention, and offer new perspectives and strategies for comprehensive myopia control in children.
Item 3. What (Materials): Description of any physical or informational materials used in the intervention, including those used during delivery, provided to participants, or used for training intervention providers; information on how to access these materials (e.g., online appendix, links).	The intervention involved two phases: an imagery encoding phase and a maintenance phase. Intervention materials included pictures and a dynamic visual task device. Training materials comprised guided imagery scripts and an imagery feedback log. The guided scripts described when, where, why, and how children should use motor imagery to improve visual function, including techniques for generating clear, vivid, and controllable imagery. The study included two groups: G1 and G2.
Item 4. What (Procedures): Description of each procedure, activity, and/or process involved in delivering the intervention, including any facilitation or supportive activities.	To ensure experimental control, all intervention and assessment personnel were graduate students specializing in exercise science and sport psychology. Training was delivered face-to-face in groups of 10 within each class. During the intervention, participants practiced general cognitive imagery, and specific cognitive imagery. Before training, children were informed about the importance of visual function and the principles of motor imagery training to enhance motivation. Different guided imagery modes were used to gradually lead children into an imagery state, encouraging them to imagine performing real visual tasks (e.g., seeing distant objects clearly or tracking a moving visual target). Attention guidance strategies were embedded in the scripts, with dedicated attention phases lasting 30–60 s. The rhythm and content of the guidance were adjusted to maintain high levels of attention.
Item 5. Who provided: Description of the expertise, background, and professional training of individuals delivering the intervention (e.g., psychologists, teachers, or assistants).	Delivered primarily by trained graduate students in exercise science and sport psychology. Facilitators received structured training from the research team in motor imagery theory, PETTLEP principles, and standardized script administration. The physical education teacher received basic training and provided supportive classroom assistance but did not lead the imagery instructions.
Item 6. How: Description of the mode of delivery (e.g., face-to-face, online, or telephone) and whether the intervention was delivered individually or in groups.	Delivered face-to-face in classroom settings in small groups of approximately 10 children. Sessions were led by trained facilitators following a standardized script, with teacher assistance for organization and supervision.
Item 7. Where: Description of the setting in which the intervention took place, including necessary infrastructure or relevant characteristics.	Training was conducted in accordance with the PETTLEP model, ensuring consistency between imagery content and the physical environment. Sessions took place at Suzhou Science and Technology Town Experimental Primary School, either in classrooms or the equipment room, in relatively quiet environments with minimal external disturbance.
Item 8. When and how much: Description of the frequency and duration of the intervention, including number of sessions, schedule, session length, intensity, or dosage.	The intervention lasted 16 weeks, with three sessions per week. Each session included 30 imagery trials. Every 2 weeks, children also performed a near–far dynamic visual task to reinforce imagery content.
Item 9. Tailoring: If the intervention was individualized, description of what was tailored, why, when, and how.	All participants received the same core training structure, including fixed session sequence, dosage, and PETTLEP-based instructional content. Limited delivery-level adaptations were permitted, including grouping based on visual and imagery ability, selection of familiar imagery scenarios, individualized visual target design, and minor adjustments to audio delivery (e.g., speech rate and tone). These adaptations did not alter the intervention structure, dosage, or core instructional content.
Item 10. Modifications: If the intervention was modified during the study, description of what was changed, why, when, and how.	Imagery materials, guided scripts, and background audio were adjusted in real time based on participant feedback and observed engagement. All changes and their rationales were documented. No core components of the intervention were modified during the study. Minor delivery-level adjustments, such as clarifications and pacing adjustments, were made when necessary without changing the intervention content, sequence, or dosage.
Item 11. Planned adherence or fidelity: If adherence or fidelity was assessed, description of how and by whom it was evaluated, and any strategies used to maintain or enhance fidelity.	After each imagery training session, participants were required to complete an imagery log. The log included the following items: (1) whether they could clearly see themselves performing the visual task; (2) whether they could imagine and feel themselves performing the visual task; (3) which parts of the guided instructions they could not imagine or feel; (4) whether they felt sleepy or had their minds wander to other thoughts; and (5) whether they experienced any physical discomfort during the process, particularly in the eyes, and the possible reasons for it.
Item 12. Actual adherence or fidelity: If assessed, description of the extent to which the intervention was delivered as planned.	Visual function, motor imagery ability, and mental chronometry were assessed before and after the intervention to further evaluate the accuracy with which children mastered the imagery practice during the training process.
Item 13. Attention training: Description of attention-focusing or attentional control components included in the intervention.	Specific attention-guidance strategies were incorporated into the guided imagery scripts, with dedicated attention phases lasting 30–60 s during the imagery process. Particular emphasis was placed on monitoring children’s attention levels throughout the session; the pace and content of the guidance were adjusted accordingly to ensure that children maintained a high level of focused attention.
Item 14. Introduction of ciliary muscle–related imagery tasks: Description of specific imagery tasks targeting ciliary muscle movement and accommodative regulation.	Before the start of training, children were provided with a detailed explanation of the importance of visual function improvement and the principles of motor imagery training to enhance their motivation to participate. Different modes of guided instructions were used to gradually lead children into an imagery state, encouraging them to imagine that they were performing real visual tasks, such as clearly seeing distant objects or tracking a moving visual target.
Item 15. Feedback and reward mechanisms: Description of feedback, reinforcement, or reward strategies used to enhance engagement and motivation.	When children successfully completed a visual task or an imagery exercise, they received immediate feedback and rewards, such as verbal praise, points, or small gifts. These forms of feedback and reinforcement were intended to enhance children’s sense of achievement and self-confidence, thereby increasing their willingness to engage in training and maintain focused attention on the visual tasks.
Item 16. Imagery training procedure: Description of the structured steps or sequence of the motor imagery training process.	Training was scheduled three times per week, with each session consisting of 30 imagery trials. This arrangement aimed to ensure training continuity while avoiding excessive practice that could lead to fatigue or resistance among participants.
Item 17. Imagery training plan: Description of the overall training schedule, progression, and organization of imagery practice.	At the beginning of the intervention, children underwent visual function testing and assessment to establish baseline levels and identify existing problems. Based on the assessment results, individualized training plans were developed, including decisions regarding training content, frequency, and intensity. A variety of training methods and approaches were employed, such as visual tracking tasks and imagery ability exercises, to comprehensively stimulate the visual system and promote visual function development.
Item 18. Evaluation and feedback: Description of methods used to evaluate training outcomes and provide feedback to participants.	The intervention was delivered according to the predefined standardized script, with consistent session structure, sequence, and dosage across groups. No modifications were made to core training components. Minor clarifications or pacing adjustments were provided when necessary without altering the intervention content. Attendance and classroom engagement were recorded to reflect adherence.

The intervention was developed following a structured protocol to ensure procedural consistency across participants and sessions. Core components of the training were fully standardized, including the overall session structure, sequence of imagery phases, training frequency, session duration, instructional logic, and adherence to PETTLEP-based principles. The scripted content guiding near–far visual transitions and ciliary muscle engagement remained identical for all groups, and no modifications were made to these core elements during the study (Item 18).

At the same time, limited delivery-level adaptations were permitted to enhance developmental appropriateness and imagery clarity without altering the core protocol (Item 9). Specifically, participants were grouped based on visual function and imagery ability to facilitate appropriate pacing and guidance. For general imagery practice, scenario materials were selected from familiar daily-life contexts and commonly participated sports activities identified through student voting, with the aim of increasing engagement and imagery vividness. For specific imagery practice, individualized visual target designs were used, and minor adjustments in audio delivery parameters (e.g., speech rate, tone, and background sound intensity) were made when necessary to optimize comprehension and imagery clarity. These adaptations were restricted to delivery format and presentation style and did not modify the training structure, dosage, or instructional content.

Thus, the intervention maintained a standardized core structure while permitting limited delivery-level adaptations to support developmental appropriateness, consistent with established principles for complex intervention development (Onken et al., 2014; Skivington et al., 2021).

## 3 Results

### 3.1 Participant flow and intraclass correlation coefficients (ICCs)

**Figure 2:**
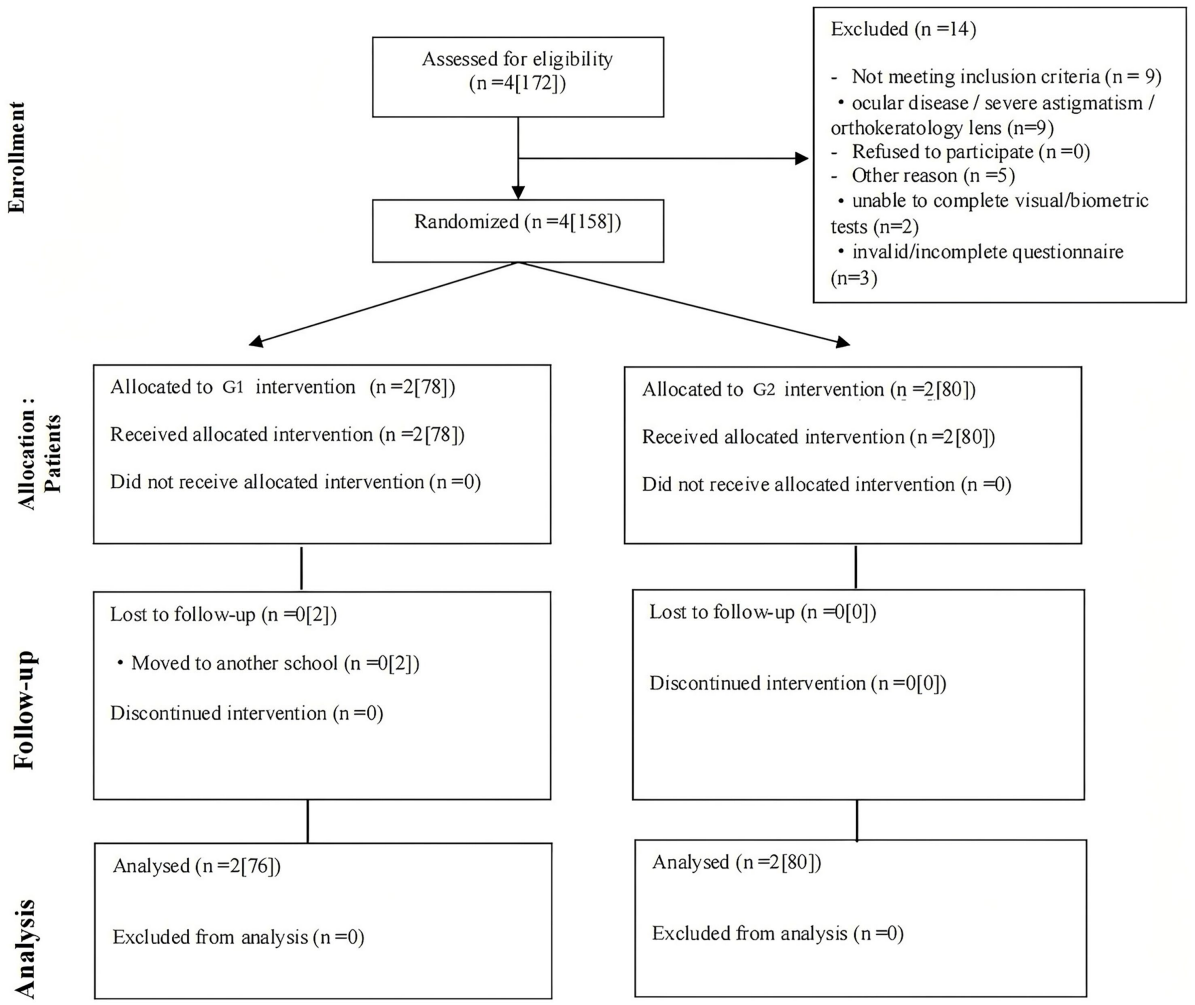
CONSORT flow diagram of classes and children through the trial. Numbers are reported as classes [children].

Participant flow is shown in Figure 2. Four intact classes were randomized, comprising 158 children (G1: 2 classes [78 children]; G2: 2 classes [80 children]). Two children in the G1 condition moved to another school and were not included in the post-intervention analysis; therefore, the primary analysis included 156 children (G1: *n* = 76; G2: *n* = 80) (Figure 2).

**Figure 3:**
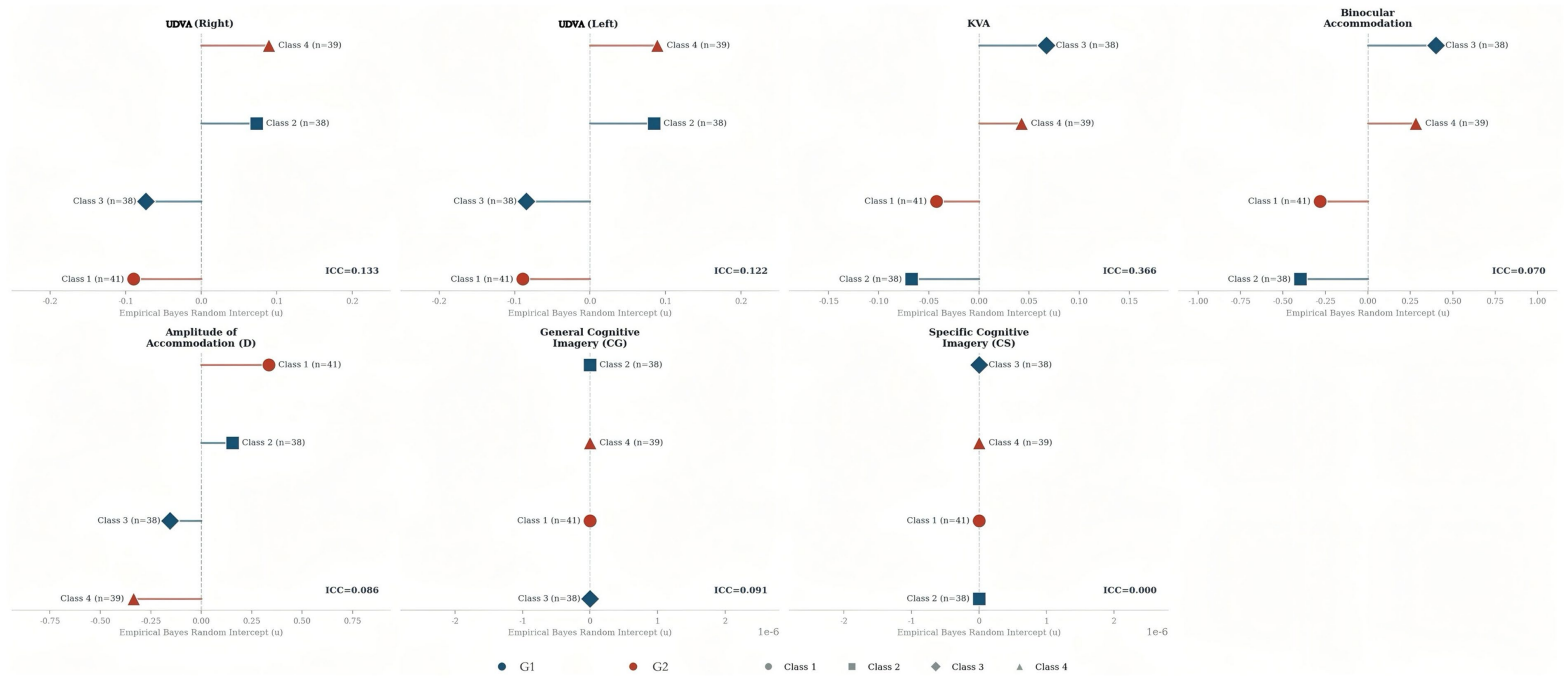
Empirical Bayes random intercept estimates by class (Marker size proportional to class size; ICC from null model).

Given the class-level allocation, class-level intraclass correlation coefficients (ICCs) were estimated from null random-intercept models to quantify within-class dependence. ICCs ranged from 0.000 to 0.366 across outcomes, indicating varying degrees of clustering. The highest ICC was observed for post-KVA (ICC = 0.366), followed by post-UDVA (right) (ICC = 0.133) and post-UDVA (left) (ICC = 0.122). Moderate ICCs were also observed for post-CG (ICC = 0.091), post-AMP (ICC = 0.086), and post-BAF (ICC = 0.071), whereas post-CS showed no detectable class-level clustering (ICC = 0.000) (Figure 3).

### 3.2 Primary outcome: UDVA

Left- and right-eye UDVA were analyzed using linear mixed-effects models with a random intercept for class and adjustment for the corresponding baseline UDVA. No between-group difference was observed at post-test for left-eye UDVA (*β =* −0.017, 95% CI [−0.270, 0.236], *p* = 0.894; BH-adjusted *p* = 0.999; Cohen’s *d* = −0.164) or for right-eye UDVA (*β =* 0.000, 95% CI [−0.237, 0.237], *p* = 0.999; BH-adjusted *p* = 0.999; Cohen’s *d* = −0.121) (Table 5).

**Table 5:** Primary outcomes: adjusted between-group differences in UDVA at T3.

Outcome	*β* (G2 vs. G1)	SE	95% CI	*t*	df	*p*	*p* (BH-adjusted)	Cohen’s *d*
UDVA (left)	−0.017	0.128	[−0.270, +0.236]	−0.134	149	0.894	0.999	−0.164
UDVA (right)	0.000	0.120	[−0.237, 0.237]	0.001	149	0.999	0.999	−0.121

β represents the adjusted between-group effect of G2 relative to G1 after controlling for the corresponding baseline covariate. 95% CI indicates the Wald confidence interval. Cohen’s *d* represents the unadjusted effect size. * indicates *p* < 0.05 after Benjamini–Hochberg (BH) correction. df = 149 refers to the residual degrees of freedom.

### 3.3 Secondary outcomes

#### 3.3.1 Visual function outcomes

Adjusted between-group comparisons at T3 for visual function outcomes are shown in Table 6. After Benjamini–Hochberg correction, KVA remained significantly different between groups (*β =* −0.252, 95% CI [−0.422, −0.082], BH-adjusted *p* = 0.014), with a large effect size (Cohen’s *d* = −1.194). Because negative *β* values indicate more favorable outcomes in the G1, this result indicates that the G1 showed better post-intervention KVA than the G2.

**Table 6:** Secondary outcomes: adjusted between-group differences at T3.

Outcome	β (G2 vs. G1)	SE	95% CI	t	df	p	p (BH-adjusted)	Cohen’s d
KVA	−0.252	0.086	[−0.422, −0.082]	−2.922	149	0.004	0.012*	−1.196
BAF	−1.285	0.741	[−2.750, 0.179]	−1.734	149	0.085	0.128	−0.410
AMP	−1.164	0.583	[−2.316, −0.012]	−1.996	149	0.048	0.096	−0.486
CG	−2.493	0.663	[−3.803, −1.183]	−3.760	149	<0.001	0.001*	−0.598
CS	0.447	0.635	[−0.807, 1.701]	0.704	149	0.483	0.579	0.114

β represents the adjusted between-group effect of G2 relative to G1 after controlling for the corresponding baseline covariate. 95% CI indicates the Wald confidence interval. Cohen’s d represents the unadjusted effect size. * indicates *p* < 0.05 after Benjamini–Hochberg (BH) correction. df = 149 refers to the residual degrees of freedom.

For BAF, the adjusted between-group difference was not statistically significant after correction (*β =* −1.285, 95% CI [−2.750, 0.179], BH-adjusted *p* = 0.149; Cohen’s *d* = −0.411). For AMP, the adjusted between-group difference also did not remain significant after correction, although a borderline trend was observed (*β =* −1.164, 95% CI [−2.316, −0.012], BH-adjusted *p* = 0.111; Cohen’s *d* = −0.486).

**Figure 4:**
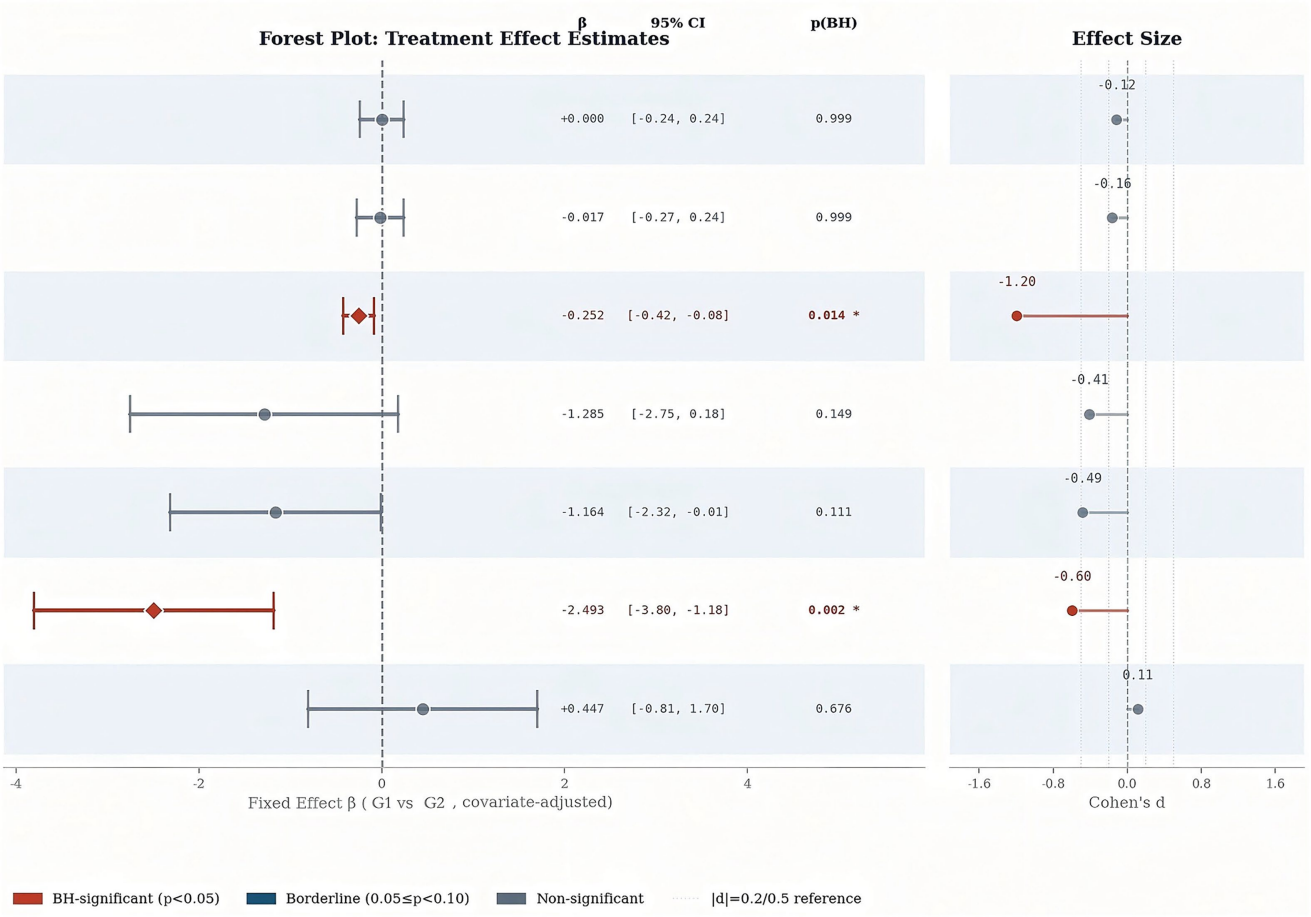
Forest plot of adjusted between-group intervention effects at post-test.

Overall, among the visual function outcomes, only KVA showed a statistically significant adjusted between-group difference after multiple-comparison correction, whereas BAF was not significant and AMP showed a borderline trend (Figure 4).

#### 3.3.2 Imagery ability outcomes

Adjusted between-group comparisons at T3 for imagery ability outcomes are also shown in Table 6. After Benjamini–Hochberg correction, CG showed a statistically significant between-group difference (*β =* −2.493, 95% CI [−3.803, −1.183], BH-adjusted *p* = 0.002), with a moderate effect size (Cohen’s d = −0.598). Because negative *β* values indicate more favorable outcomes in the G1, this finding indicates that the G1 achieved higher post-intervention CG scores than the G2.

In contrast, cognitive specific imagery (CS) did not differ significantly between groups after correction (*β =* 0.447, 95% CI [−0.807, 1.701], BH-adjusted *p* = 0.676; Cohen’s d = 0.114), indicating no clear between-group difference in this outcome.

Overall, among the imagery ability outcomes, only CG remained significantly different between groups after multiple-comparison correction, whereas CS showed no significant adjusted between-group difference.

### 3.4 Longitudinal and interaction analyses

**Figure 5:**
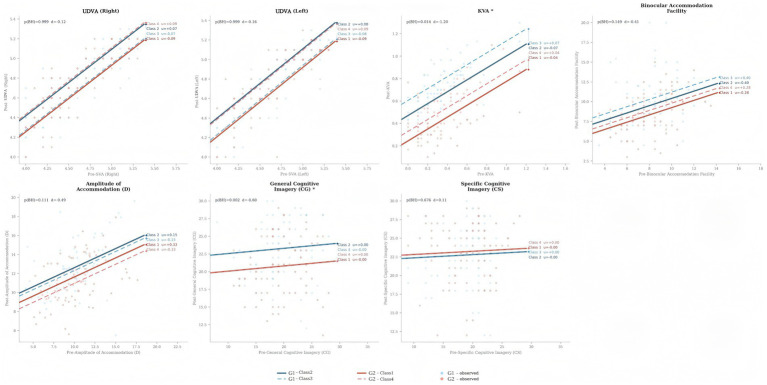
Spaghetti plots with cluster-specific fitted lines.

To complement the adjusted between-group comparisons at T3, additional linear mixed-effects analyses were conducted to examine within-group time effects and Time × Group interactions (Table 7 and Figure 5).

**Table 7:** Within-group time effects and Time × Group interactions from linear mixed-effects models.

Outcome	G1 time effect				G2 time effect				Time × Group interaction			
	*β*	95% CI	*p*	*d*	*β*	95% CI	*p*	*d*	*β*	95% CI	*p*	*p* (BH-adjusted)
UDVA (left)	+0.114	[+0.003, +0.226]	0.044*	+0.591	+0.109	[−0.004, +0.223]	0.058	+0.500	−0.005	[−0.164, +0.153]	0.950	0.950
UDVA (right)	+0.134	[+0.018, +0.251]	0.024*	+0.647	+0.150	[+0.041, +0.259]	0.007*	+0.743	+0.016	[−0.143, +0.175]	0.845	0.950
KVA	+0.379	[+0.308, +0.450]	<0.001*	+2.174	+0.119	[+0.046, +0.192]	0.002*	+0.542	−0.260	[−0.361, −0.159]	<0.001	<0.001*
BAF	+1.974	[+1.101, +2.847]	<0.001*	+0.592	+0.563	[−0.167, +1.292]	0.130	+0.220	−1.411	[−2.541, −0.281]	0.015	0.034*
AMP	+2.255	[+1.374, +3.135]	<0.001*	+0.794	+1.190	[+0.367, +2.013]	0.005*	+0.423	−1.065	[−2.266, +0.137]	0.082	0.144
CG	+4.092	[+2.707, +5.478]	<0.001*	+0.688	+1.362	[+0.072, +2.653]	0.039*	+0.246	−2.730	[−4.613, −0.846]	0.005	0.016*
CS	+3.789	[+2.595, +4.984]	<0.001*	+0.757	+4.163	[+2.863, +5.462]	<0.001*	+0.708	+0.373	[−1.389, +2.135]	0.677	0.948

*β* values for the G1 and G2 time effects were estimated from separate linear mixed-effects models fitted within each group using restricted maximum likelihood (REML). The interaction estimate (β_inter) was obtained from the combined model; negative values indicate greater improvement in G1 than in G2. *p*(BH) denotes the Benjamini–Hochberg-adjusted p value for the interaction terms across the seven outcomes. *d* represents Cohen’s *d* for paired designs. * indicates *p* < 0.05. For CG and CS, the “Pre→Post” comparison refers to the contrast between T1 and T3.

Within-group analyses showed that the G1 improved significantly over time in left-eye UDVA (*β =* 0.114, 95% CI [0.003, 0.226], *p* = 0.044), right-eye UDVA (*β =* 0.134, 95% CI [0.018, 0.251], *p* = 0.024), KVA (*β =* 0.379, 95% CI [0.308, 0.450], *p <* 0.001), BAF (*β =* 1.974, 95% CI [1.101, 2.847], *p <* 0.001), AMP (*β =* 2.255, 95% CI [1.374, 3.135], *p <* 0.001), CG (*β =* 4.092, 95% CI [2.707, 5.478], *p <* 0.001), and CS (*β =* 3.789, 95% CI [2.595, 4.984], *p <* 0.001).

In the G2, significant within-group improvements were observed for right-eye UDVA (*β =* 0.150, 95% CI [0.041, 0.259], *p* = 0.007), KVA (*β =* 0.119, 95% CI [0.046, 0.192], *p* = 0.002), AMP (*β =* 1.190, 95% CI [0.367, 2.013], *p* = 0.005), CG (*β =* 1.362, 95% CI [0.072, 2.653], *p* = 0.039), and CS (*β =* 4.163, 95% CI [2.863, 5.462], *p <* 0.001), whereas changes in left-eye UDVA and BAF did not reach statistical significance.

For the Time × Group interaction, significant differential change over time was observed for KVA (*β =* −0.260, 95% CI [−0.361, −0.159], BH-adjusted *p <* 0.001), BAF (*β =* −1.411, 95% CI [−2.541, −0.281], BH-adjusted *p* = 0.034), and CG (*β =* −2.730, 95% CI [−4.613, −0.846], BH-adjusted *p* = 0.016), indicating that the magnitude of improvement differed between groups for these outcomes. No significant interaction was found for left-eye UDVA, right-eye UDVA, AMP, or CS after correction (all BH-adjusted *p >* 0.05).

Overall, these complementary analyses indicated that both groups improved over time on several outcomes. The G1 showed clearer advantages for CG, whereas the observed KVA pattern should be interpreted cautiously as a class-level observation given the limited number of clusters and the relatively high ICC for that outcome.

### 3.5 Simple-effect and pairwise analyses

The simple effects analysis showed that the post-test group differences for KVA and CG were significant (*p <* 0.01). Children in the G1 had significantly higher KVA and CG compared to the G2. The time effects showed that children in the G2 had significantly higher KVA in the post-test compared to the pre-test (*p <* 0.01); and the post-test CG was significantly higher than both the mid-test and pre-test (*p <* 0.05). In the G1, children showed significantly higher KVA in the post-test compared to the pre-test (*p <* 0.01); post-test CG was higher than pre-test (*p <* 0.01) and mid-test (*p <* 0.05), and the mid-test was higher than the pre-test (*p <* 0.05) (Table 8).

**Table 8:** Simple effects and time point change analysis.

Outcome	Group	Comparison	n	Effect Size (Δ)	95% CI	t	df	p	p (BH-adjusted)	Cohen’s d
UDVA (left)	G1	Post−Pre	76	+0.134	[+0.087, +0.182]	5.637	75	<0.001	<0.001*	+0.647
	G2	Post−Pre	80	+0.150	[+0.105, +0.195]	6.645	79	<0.001	<0.001*	+0.743
	G2 − G1	ΔG2 − ΔG1	156	+0.018	[−0.185, +0.221]	0.171	150	0.864	0.980	+0.077
UDVA (right)	G1	Post−Pre	76	+0.114	[+0.070, +0.159]	5.152	75	<0.001	<0.001*	+0.591
	G2	Post−Pre	80	+0.109	[+0.061, +0.158]	4.469	79	<0.001	<0.001*	+0.500
	G2 − G1	ΔG2 − ΔG1	156	−0.003	[−0.237, +0.231]	−0.025	150	0.980	0.980	−0.025
KVA	G1	Post−Pre	76	+0.379	[+0.339, +0.419]	18.955	75	<0.001	<0.001*	+2.174
	G2	Post−Pre	80	+0.119	[+0.070, +0.168]	4.846	79	<0.001	<0.001*	+0.542
	G2 − G1	ΔG2 − ΔG1	156	−0.259	[−0.447, −0.071]	−2.722	150	0.007	0.044*	−1.308
BAF	G1	Post−Pre	76	+1.974	[+1.212, +2.736]	5.160	75	<0.001	<0.001*	+0.592
	G2	Post−Pre	80	+0.563	[−0.007, +1.132]	1.965	79	0.053	0.053	+0.220
	G2 − G1	ΔG2 − ΔG1	156	−1.401	[−2.967, +0.164]	−1.769	150	0.079	0.184	−0.476
AMP	G1	Post−Pre	76	+2.255	[+1.606, +2.903]	6.924	75	<0.001	<0.001*	+0.794
	G2	Post−Pre	80	+1.190	[+0.564, +1.816]	3.786	79	<0.001	<0.001*	+0.423
	G2 − G1	ΔG2 − ΔG1	156	−1.077	[−2.603, +0.448]	−1.395	150	0.165	0.289	−0.377
CG	G1	Pre − Post	76	+4.092	[+2.732, +5.452]	5.994	75	<0.001	<0.001*	+0.688
	G1	Pre → Mid	76	+3.289	[+2.042, +4.537]	5.252	75	<0.001	<0.001*	+0.602
	G1	Mid → Post	76	+0.803	[+0.085, +1.520]	2.228	75	0.029	0.045*	+0.256
	G2	Post−Pre	80	+1.363	[+0.131, +2.594]	2.202	79	0.031	0.033*	+0.246
	G2	Pre → Mid	80	+0.450	[−0.508, +1.408]	0.935	79	0.353	0.385	+0.105
	G2	Mid → Post	80	+0.912	[+0.071, +1.754]	2.158	79	0.034	0.045*	+0.241
	G2 − G1	ΔG2 − ΔG1	156	−2.725	[−4.854, −0.595]	−2.528	150	0.013	0.044*	−0.475
CS	G1	Pre − Post	76	+3.789	[+2.646, +4.933]	6.601	75	<0.001	<0.001*	+0.757
	G1	Pre → Mid	76	+0.684	[−0.219, +1.588]	1.509	75	0.136	0.163	+0.173
	G1	Mid → Post	76	+3.105	[+2.314, +3.896]	7.818	75	<0.001	<0.001*	+0.897
	G2	Post−Pre	80	+4.162	[+2.855, +5.470]	6.335	79	<0.001	<0.001*	+0.708
	G2	Pre → Mid	80	+4.100	[+2.809, +5.391]	6.321	79	<0.001	<0.001*	+0.707
	G2	Mid → Post	80	+0.063	[−0.908, +1.033]	0.128	79	0.898	0.898	+0.014
	G2 − G1	ΔG2 − ΔG1	156	+0.373	[−1.358, +2.104]	0.426	150	0.671	0.939	+0.068

Within-group Δ = Post − Pre, one-sample t-test (H₀: Δ = 0); Between-group ΔΔ = ΔG2 − ΔG1, LMM estimation (random intercept by class); Pairwise comparison of CG/CS is performed using paired *t*-test. BH correction is independently executed for each group and each outcome across 3 comparisons. *d* = paired Cohen’s *d*; * indicates *p* < 0.05 (within-group/between-group delta) or *p* < 0.05 (pairwise comparison).

## 4 Discussion

The present study had two aims: to develop a ciliary muscle-oriented motor imagery (MI) script for primary school children based on the PETTLEP model, and to compare two school-based training arrangements, namely staged imagery (G1) and specific-only imagery (G2). Overall, both arrangements were associated with improvements over time in several outcomes. The clearest between-group evidence was observed for imagery-related performance, particularly CG, which favored G1. By contrast, the visual-function findings were more limited, and the between-group KVA result should be interpreted cautiously given the small number of clusters and the relatively high class-level ICC.

### 4.1 Visual-function changes associated with different motor imagery training arrangements

Among the visual-function outcomes, KVA was the only measure that showed a statistically significant adjusted between-group difference at post-test. By contrast, no between-group difference was observed for UDVA, whereas BAF was not significant after correction and AMP showed only a borderline trend. However, the KVA finding should be interpreted cautiously because the study included only four classes in total, with two classes per condition, and the class-level ICC for post-intervention KVA was relatively high (ICC = 0.366). Under these conditions, the observed KVA difference is better regarded as a hypothesis-generating class-level observation than as reliable evidence of a differential training effect.

Although G1 showed a numerically more favorable KVA pattern than G2, several factors may help explain this pattern. First, primary school children are in a developmental period in which visual–motor integration remains highly malleable. Pediatric visual-motor research suggests that late childhood to early adolescence may represent a sensitive period for the refinement of dynamic visual tracking and related perceptual skills (e.g., 8–14 years), during which training effects may be amplified by ongoing neuroplastic development (Yin et al., 2023). Second, the relatively low baseline KVA level in the present sample may have left substantial room for measurable improvement. Third, although conventional physical or device-based visual training often yields the clearest gains in accommodative regulation, the present MI protocol may also have engaged broader visual-motor processes, such as attentional allocation and motion prediction, through repeated imagery of near-far switching and moving-target tracking. This selective pattern is partly consistent with our previous school-based study, in which imagery-integrated training was also associated with improvements in dynamic visual outcomes, whereas changes across visual indicators were not entirely uniform.

Our previous studies found that children sometimes struggled to imagine visual targets that were unfamiliar in daily life, which may have constrained training outcomes. From this perspective, general imagery may have provided a more accessible entry point by helping children build a clearer imagery framework before progressing to more specific visual tasks. Because general imagery is grounded in everyday contexts, it may also have enhanced children’s sensitivity to visual-adjustment experiences in real-life situations, thereby supporting subsequent task-specific imagery, although this process was not directly measured in the present study.

Existing MI interventions vary considerably in their design, implementation methods, and target populations (Williams et al., 2013a). Therefore, the present findings are best interpreted as suggesting that a staged sequence involving general imagery followed by specific imagery may be associated with a different developmental pattern from specific imagery alone, rather than as confirming a unique preparatory effect of general imagery. More rigorous studies with additional clusters per condition and stronger control designs are needed to determine whether this pattern can be replicated.

### 4.2 Imagery-ability changes associated with different motor imagery training arrangements

The present imagery-ability findings should be interpreted in light of the unique characteristics of ciliary muscle-oriented imagery. Unlike skeletal muscles, the ciliary muscle is a small intraocular smooth muscle whose activity is neither externally visible nor accompanied by direct conscious proprioceptive feedback. Accordingly, the kinesthetic component in the present study should not be understood as direct perception of a specific muscle state, but rather as the subjective re-experiencing of visual-adjustment-related sensations, such as changes in visual clarity, ocular effort, and ocular comfort. Although the ciliary muscle has traditionally been regarded as difficult to regulate voluntarily because of its autonomic innervation, some previous findings suggest that accommodative regulation may, to a limited extent, be influenced by conscious processes (Sisson, 1937; Campbell, 1959; Wagner et al., 2019). Biofeedback research has also shown that participants can learn to relax and contract accommodation under training conditions (Trachtman et al., 1981). Related studies further indicate that imagery may affect autonomically regulated ocular responses, including pupil-size changes during imagined luminance variation (Laeng and Teodorescu, 2002), and may even be associated with lens-curvature changes during imagery of objects at different distances (Ruggieri and Alfieri, 1992). These findings provide a tentative rationale for exploring imagery-based approaches in visual-function interventions, although direct causal evidence remains limited.

Imagery ability refers to an individual’s capacity to generate, maintain, and manipulate mental images, and it is an important determinant of the effectiveness of MI interventions (Kraeutner et al., 2020). In the CG dimension, the G1 scored significantly higher than the G2 at both the mid- and post-tests, with a generally increasing trend over time. This pattern is broadly consistent with previous imagery research suggesting that imagery ability is an important determinant of intervention responsiveness and that more accessible, context-rich imagery may facilitate later task-specific imagery performance (Kraeutner et al., 2020). One possible interpretation is that the first eight weeks of general imagery training, which relied on natural scenes and everyday-life materials, helped children develop greater subjective clarity and perceived control over their imagery. Such staged exposure may have been particularly beneficial for children with relatively weak baseline imagery ability, although this possibility requires direct examination in future studies.

In the CS dimension, both groups improved over time, and by the later stage of training, children in the G1 reached a level comparable to that of the G2. This convergence partly echoes previous work indicating that different imagery formats may support skill development through different pathways, even when later-stage performance becomes comparable. This pattern is consistent with the possibility that earlier general imagery practice helped establish a more stable representational framework, which may have reduced the cognitive effort required during later specific imagery tasks. However, because cognitive load and attentional allocation were not directly measured, the current design does not allow firm conclusions regarding the unique preparatory role of general imagery.

It is also noteworthy that the G2’s CS scores increased markedly from T1 to T2 but showed minimal change from T2 to T3, indicating a plateau in the later stage of measurement. Several explanations may account for this pattern. First, because the SIQ subscale has a limited scoring range, relatively high mid-intervention scores may have reduced the remaining measurable room for improvement, suggesting a potential ceiling effect. Second, repeated administration of the same self-report questionnaire may have led to response stabilization or habituation as children became more familiar with the items. Third, because the SIQ relies on self-reported imagery experience, social desirability or expectancy-related responding cannot be fully excluded in classroom-based intervention contexts. Future studies may therefore benefit from incorporating more sensitive multi-method assessments of imagery ability, such as behavioral imagery tasks or objective physiological indices.

## 5 Conclusion and future directions

### 5.1 Conclusion

This study developed and preliminarily evaluated a ciliary muscle-oriented motor imagery (MI) script for primary school children under school-based conditions. The findings suggest that different imagery arrangements may be associated with different developmental patterns, with the clearest between-group evidence observed for cognitive-general imagery. Visual-function findings were more limited, and the KVA result in particular should be interpreted cautiously. Further studies with more clusters, stronger control conditions, and longer follow-up are needed to confirm these findings and clarify the mechanisms involved.

### 5.2 Future directions

First, future studies should use more rigorous multi-arm designs to disentangle the respective contributions of imagery content and training sequence. In addition to G1 and G2 conditions, designs incorporating non-MI controls, alternative active controls, or reversed sequencing conditions (e.g., specific imagery followed by general imagery) would help clarify whether the observed differences are attributable to general imagery, sequencing effects, or their combination.

Second, future work should incorporate more direct process measures to test the interpretations proposed in the present study. Measures of imagery vividness, controllability, cognitive load, and attentional allocation would help determine whether staged imagery improves children’s engagement with the intervention by reducing early task difficulty or by strengthening representational stability.

Third, future research should include more sensitive and multimethod assessments of both visual and imagery-related outcomes. In addition to self-report measures such as the SIQ, behavioral imagery tasks, dynamic accommodation assessments, ciliary muscle imaging, or other physiological indicators may provide more direct evidence regarding the mechanisms underlying imagery-based visual-function interventions.

Finally, larger multicenter school-based studies with longer follow-up periods are needed to evaluate generalizability, maintenance of effects, and practical applicability across different educational contexts. Such work would help determine whether staged MI training can serve as a sustainable complementary component of children’s visual-health support programs.

## 6 Limitations

Several limitations should be acknowledged when interpreting the present findings.

First, because both groups received MI training and no passive or alternative active control condition was included, the observed improvements cannot be attributed specifically to MI. Developmental maturation, repeated-measurement practice effects, expectancy influences, and the non-specific benefits of participating in structured classroom activities may also have contributed to the observed changes.

Second, the two intervention conditions differed simultaneously in both imagery content and training sequence. Therefore, any observed advantage of G1 cannot be attributed specifically to the preparatory role of general imagery, content differences, or sequencing effects alone.

Third, imagery vividness, controllability, cognitive load, and attentional allocation during training were not directly measured, so the proposed explanatory mechanisms remain tentative.

Fourth, imagery ability was assessed primarily through self-report. Although the adapted SIQ showed acceptable psychometric support in our previous work, repeated self-report measurement in children may still be influenced by limited introspective accuracy, response habituation, expectancy effects, and possible ceiling effects, particularly when interpreting the late-stage plateau in G2’s CS scores.

Finally, the clustered design imposes an important limitation on the interpretation of the visual-function findings, especially KVA. The study included only four classes from a single school, with two classes per condition, and the class-level ICC for post-intervention KVA was relatively high (ICC = 0.366). Accordingly, the observed between-group KVA difference should be regarded as a hypothesis-generating class-level observation rather than confirmatory evidence of a reliable training effect. Replication in larger and more diverse school-based samples with more clusters per condition will be necessary to establish the robustness and generalizability of the present findings.

## Data Availability

The raw data supporting the conclusions of this article will be made available by the authors, without undue reservation.

## References

[ref1] Baird P. N. Saw S. M. Lanca C. Guggenheim J. A. Smith E. L. Zhou X. . (2020). Myopia. Nat. Rev. Dis. Prim. 6:99. doi: 10.1038/s41572-020-00231-4 33328468

[ref9001] Billot L. Copas A. Leyrat C. Forbes A. Turner E. L. (2024). How should a cluster randomized trial be analyzed?. Journal of epidemiology and population health, 72:202196. 38477477 10.1016/j.jeph.2024.202196 PMC7616648

[ref9002] Burns D. H. Allen P. M. Edgar D. F. Evans B. J. (2020). Sources of error in clinical measurement of the amplitude of accommodation. Journal of Optometry, 13, 3–14. 31303551 10.1016/j.optom.2019.05.002 PMC6951837

[ref2] Campbell F. W. (1959). The accommodation response of the human eye. Br. J. Physiol. Opt. 16, 188–203, 13807257

[ref3] Chassine T. Villain M. Hamel C. P. Daien V. (2015). How can we prevent myopia progression? Eur. J. Ophthalmol. 25, 280–285. doi: 10.5301/ejo.5000571 25655598

[ref4] Cumming J. Bird G. Brown K. Kolitsida M. Quinton M. (2023). “Imagery,” in Routledge Handbook of Applied sport Psychology: A Comprehensive guide for Students and Practitioners, eds. Tod D. Hodge K. Krane V. (London: Routledge), 543–552.

[ref5] Cumming J. Quinton M. L. (2022). Improving the reporting of sport imagery interventions with TIDieR. Asian J. Sport Exerc. Psychol. 2, 80–90. doi: 10.1016/j.ajsep.2022.07.003

[ref6] Currie G. (1995). Visual imagery as the simulation of vision. Mind Lang. 10, 25–44. doi: 10.1111/j.1468-0017.1995.tb00004.x

[ref7] Dijkers M. P. (2021). Overview of reviews using the template for intervention description and replication (TIDieR) as a measure of trial intervention reporting quality. Arch. Phys. Med. Rehabil. 102, 1623–1632. doi: 10.1016/j.apmr.2020.09.397, 33245937

[ref8] Flügel C. Bárány E. H. Lütjen-Drecoll E. (1990). Histochemical differences within the ciliary muscle and its function in accommodation. Exp. Eye Res. 50, 219–226. doi: 10.1016/0014-4835(90)90234-L 2138092

[ref9] Fusi S. Cutuli D. Valente M. R. Bergonzi P. Porro C. A. Di Prampero P. E. (2005). Cardioventilatory responses during real or imagined walking at low speed. Arch. Ital. Biol. 143, 223–228, 16097499

[ref10] Gupta S. Joshi A. Saxena H. Chatterjee A. (2021). Outdoor activity and myopia progression in children: a follow-up study using mixed-effects model. Indian J. Ophthalmol. 69, 3446–3450. doi: 10.4103/ijo.IJO_3602_20, 34826972 PMC8837331

[ref11] Holmes P. S. Collins D. J. (2001). The PETTLEP approach to motor imagery: a functional equivalence model for sport psychologists. J. Appl. Sport Psychol. 13, 60–83. doi: 10.1080/10413200109339004

[ref12] Huang Y. Li M. Shen Y. Liu F. Fang Y. Xu H. . (2022). Study of the immediate effects of autostereoscopic 3D visual training on the accommodative functions of myopes. Investig. Ophthalmol. Vis. Sci. 63:9. doi: 10.1167/iovs.63.2.9 PMC8819359 35113140

[ref13] Kosslyn S. M. Shwartz S. P. (1977). A simulation of visual imagery. Cogn. Sci. 1, 265–295. doi: 10.1016/S0364-0213(77)80020-7

[ref14] Kraeutner S. N. Eppler S. N. Stratas A. Boe S. G. (2020). Generate, maintain, manipulate? Exploring the multidimensional nature of motor imagery. Psychol. Sport Exerc. 48:101673. doi: 10.1016/j.psychsport.2020.101673

[ref15] Laeng B. Sulutvedt U. (2014). The eye pupil adjusts to imaginary light. Psychol. Sci. 25, 188–197. doi: 10.1177/0956797613503556, 24285432

[ref16] Laeng B. Teodorescu D. S. (2002). Eye scanpaths during visual imagery reenact those of perception of the same visual scene. Cogn. Sci. 26, 207–231. doi: 10.1207/s15516709cog2602_3

[ref17] Liu M. (2001). Discussion on the new procedure of mental imagery training. Psychol. Sci. 2:132-134+252. doi: 10.16719/j.cnki.1671-6981.2001.02.002

[ref18] Lu J. Zhao A. H. Wang C. Y. Wang H. R. Gu C. Y. Lu L. Y. (2021). Effect of visual training combined with anti-peripheral hyperopia optical defocus lens on myopia control in adolescents. Chin. J. Ophthalmol. Tradit. Chinese Med. 31, 566–569. doi: 10.13444/j.cnki.zgzyykzz.2021.08.007

[ref9003] Momeni-Moghaddam H. Kundart J. Askarizadeh F. (2014). Comparing measurement techniques of accommodative amplitudes. Indian Journal of Ophthalmology, 62, 683–687. 25005195 10.4103/0301-4738.126990 PMC4131318

[ref19] Munzert J. Lorey B. Zentgraf K. (2009). Cognitive motor processes: the role of motor imagery in the study of motor representations. Brain Res. Rev. 60, 306–326. doi: 10.1016/j.brainresrev.2008.12.024, 19167426

[ref20] Narayanasamy S. Vincent S. J. Sampson G. P. Wood J. M. (2016). Visual demands in modern Australian primary school classrooms. Clin. Exp. Optom. 99, 233–240. doi: 10.1111/cxo.12365, 26889920

[ref21] Onken L. S. Carroll K. M. Shoham V. Cuthbert B. N. Riddle M. (2014). Reenvisioning clinical science: unifying the discipline to improve the public health. Clin. Psychol. Sci. 2, 22–34. doi: 10.1177/2167702613497932, 25821658 PMC4374633

[ref22] Ovenseri-Ogbomo G. O. Oduntan O. A. (2015). Mechanism of accommodation: a review of theoretical propositions. African Vis. Eye Heal. 74:a28. doi: 10.4102/aveh.v74i1.28

[ref23] Paivio A. (2008). Mental Representations: A Dual Coding Approach. Oxford: Oxford University Press.

[ref24] Pearson J. Westbrook F. (2015). Phantom perception: voluntary and involuntary nonretinal vision. Trends Cogn. Sci. 19, 278–284. doi: 10.1016/j.tics.2015.03.004, 25863415

[ref25] Recko M. Stahl E. D. (2015). Childhood myopia: epidemiology, risk factors, and prevention. Mo. Med. 112, 116–121, 25958656 PMC6170055

[ref26] Rozado D. Lochner M. Engelke U. Dünser A. (2019). Detecting intention through motor-imagery-triggered pupil dilations. Hum. Comput. Interact. 34, 83–113. doi: 10.1080/07370024.2017.1293540

[ref27] Ruggieri V. Alfieri G. (1992). The eyes in imagery and perceptual processes: first remarks. Percept. Mot. Skills 75, 287–290. doi: 10.2466/pms.1992.75.1.287, 1528683

[ref28] Schachar R. A. (2006). The mechanism of accommodation and presbyopia. Int. Ophthalmol. Clin. 46, 39–61. doi: 10.1097/00004397-200604630-00006 16929224

[ref29] Sisson E. D. (1937). A case of voluntary control of accommodation. J. Gen. Psychol. 17, 170–174. doi: 10.1080/00221309.1937.9917986

[ref30] Skivington K. Matthews L. Simpson S. A. Craig P. Baird J. Blazeby J. M. . (2021). A new framework for developing and evaluating complex interventions: update of Medical Research Council guidance. BMJ 374:n2061. doi: 10.1136/bmj.n2061, 34593508 PMC8482308

[ref31] Smith E. E. (2007). Cognitive Psychology: Mind and Brain. London: Sage.

[ref32] Tao F. B. (2023). Special interpretation of“technical guidelines for comprehensive public health intervention in prevention and control of myopia in children and adolescents”. Sch. Heal. China 44, 1445–1449. doi: 10.16835/j.cnki.1000-9817.2023.10.002

[ref33] Teng Y. K. Chang C. W. Lee S. D. (2022). A multi-component physiotherapeutic intervention among schoolchildren with myopia: 3d-based vision training program with auditory frequency entrainment and electrical stimulation. Appl. Sci. 12:201. doi: 10.3390/app12010201

[ref34] Trachtman J. N. Giambalvo V. Feldman J. (1981). Biofeedback of accommodation to reduce functional myopia. Biofeedback Self Regul. 6, 547–564. doi: 10.1007/BF00998739, 7326275

[ref35] Wagner S. Zrenner E. Strasser T. (2019). Emmetropes and myopes differ little in their accommodation dynamics but strongly in their ciliary muscle morphology. Vis. Res. 163, 42–51. doi: 10.1016/j.visres.2019.08.002, 31401218

[ref9004] Wang B. Harhay M. O. Tong J. Small D. S. Morris T. P. Li F. (2026). On the mixed-model analysis of covariance in cluster-randomized trials. Statistical science: a review journal of the Institute of Mathematical Statistics 41:49. 41541796 10.1214/24-sts944 PMC12803445

[ref36] Weinberg R. (2008). Does imagery work? Effects on performance and mental skills. J. Imag. Res. Sport Phys. Act. 3:1025. doi: 10.2202/1932-0191.1025

[ref37] Williams S. E. Burns V. E. Cumming J. (2013a). Methodological variations in guided imagery interventions using movement imagery scripts in sport: a systematic review. J. Imag. Res. Sport Phys. Act. 8, 13–34. doi: 10.1515/jirspa-2012-0005

[ref38] Williams S. E. Cooley S. J. Newell E. Weibull F. Cumming J. (2013b). Seeing the difference: developing effective imagery scripts for athletes. J. Sport Psychol. Action 4, 109–121. doi: 10.1080/21520704.2013.781560

[ref39] Yin R. Zhu G. Liu A. Wang M. Li L. Dai S. (2023). The impact of additional visual tasks in physical exercise on balance ability among 9-10-year-old children: the mediating effect of visual acuity. Front. Public Health 11:1270947. doi: 10.3389/fpubh.2023.1270947, 38259731 PMC10801176

[ref40] Yusuf M. Wang N. L. (2020). Vision 2020: Progress in prevention and treatment of blindness and eye health in China. Chinese J. Med. 100, 3831–3834. doi: 10.3760/cma.j.cn112137-20200825-02468 33371626

[ref41] Zhou S. Ren Y. Ma S. Zhang M. Yin R. (2025). Improving children’s visual health by integrating motor imagery training into physical education classes. Front. Psychol. 16:1587481. doi: 10.3389/fpsyg.2025.1587481, 40552201 PMC12184536

[ref42] Zhou X. T. Wang X. Y. Qu X. M. Xu Y. Chen Z. Zhou J. Q. . (2023). Guidance of experts on eye health management of electronic screen for school-age children and adolescents. Chin. J. Ophthalmol. Otolaryngol. 23, 191–195. doi: 10.14166/j.issn.1671-2420.2023.03.001

