# Supplementary material for: Development and preliminary evaluation of a ciliary muscle–oriented motor imagery script for primary school children: a school-based cluster randomized controlled trial
Source: Front Psychol. 2026 May 7;17:1809541. doi: 10.3389/fpsyg.2026.1809541 (PMC13189959; doi:10.3389/fpsyg.2026.1809541)
Supplement: Supplementary file 1 [file Supplementary_file_1.docx]

Supplementary Material

**Supplemental Table 1.** Class-level intraclass correlation coefficients (ICCs) estimated from null mixed models.

| **Outcome** | **σ²_cluster** | **σ²_residual** | **ICC** |
| --- | --- | --- | --- |
| Post-UDVA(Left) | 0.0138 | 0.0999 | 0.122 |
| Post-UDVA(Right) | 0.0142 | 0.0927 | 0.133 |
| Post-KVA | 0.0228 | 0.0395 | 0.366 |
| Post-BAF | 0.6233 | 8.2207 | 0.071 |
| Post-AMP (D) | 0.5807 | 6.1601 | 0.086 |
| Post-Cognitive General Imagery (CG) | 1.7223 | 17.2319 | 0.091 |
| Post-Cognitive Specific Imagery (CS) | 0.0000 | 15.5674 | 0.000 |


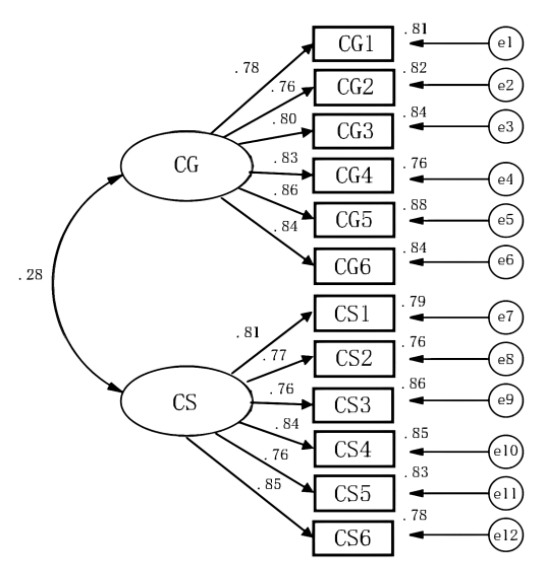


**Supplementary Figure 1.**Confirmatory factor analysis of the two-factor structure of the Sport Imagery Questionnaire (SIQ) in the present sample.
